# Minimally Invasive Tissue Sampling: A Tool to Guide Efforts to Reduce AIDS-Related Mortality in Resource-Limited Settings

**DOI:** 10.1093/cid/ciab789

**Published:** 2021-12-15

**Authors:** Emilio Letang, Natalia Rakislova, Miguel J Martinez, Juan Carlos Hurtado, Carla Carrilho, Rosa Bene, Inacio Mandomando, Llorenç Quintó, Tacilta Nhampossa, Valéria Chicamba, Elvira Luis, Mamudo R Ismail, Fabiola Fernandes, Cesaltina Lorenzoni, Luiz Ferreira, Monique Freire, Maria Teresa Rodrigo-Calvo, José Guerrero, Khátia Munguambe, Maria Maixenchs, Mireia Navarro, Isaac Casas, Lorena Marimon, Melania Ferrando, Eusebio Macete, Marcus Lacerda, Quique Bassat, Clara Menéndez, Jaume Ordi

**Affiliations:** 1 ISGlobal, Barcelona Institute for Global Health, Hospital Clínic-Universitat de Barcelona, Barcelona, Spain; 2 Department of Infectious Diseases, Hospital del Mar, Hospital del Mar Research Institute, Barcelona, Spain; 3 Department of Pathology, Hospital Clínic, Universitat de Barcelona, Spain; 4 Department of Microbiology, Hospital Clínic, Universitat de Barcelona, Spain; 5 Faculty of Medicine, Eduardo Mondlane University, Maputo, Mozambique; 6 Department of Pathology, Maputo Central Hospital, Maputo, Mozambique; 7 Department of Medicine, Maputo Central Hospital, Maputo, Mozambique; 8 Centro de Investigação em Saúde de Manhiça, Maputo, Mozambique; 9 Instituto Nacional de Saúde, Ministério da Saúde, Maputo, Mozambique; 10 Department of Pediatrics, Maputo Central Hospital, Maputo, Mozambique; 11 Department of Obstetrics and Gynecology, Maputo Central Hospital, Maputo, Mozambique; 12 Fundação de Medicina Tropical Dr. Heitor Viera Dourado, Manaus, Amazonas, Brazil; 13 Fundação Centro de Controle de Oncologia do Amazonas, Manaus, Amazonas, Brazil; 14 Institució Catalana de Recerca i Estudis Avançats (ICREA), Barcelona, Spain; 15 Pediatrics Department, Hospital Sant Joan de Déu, Universitat de Barcelona, Esplugues, Barcelona, Spain; 16 Consorcio de Investigación Biomédica en Red de Epidemiología y Salud Pública, Madrid, Spain

**Keywords:** minimally invasive autopsy, minimally invasive tissue sampling, HIV, low- and -middle-income countries

## Abstract

**Background:**

Available information on the causes of death among people living with human immunodeficiency virus (PLHIV) in low- and middle-income countries (LMICs) remains scarce. We aimed to provide data on causes of death in PLHIV from two LMICs, Brazil and Mozambique, to assess the impact of clinical misdiagnosis on mortality rates and to evaluate the accuracy of minimally invasive tissue sampling (MITS) in determining the cause of death in PLHIV.

**Methods:**

We performed coupled MITS and complete autopsy on 164 deceased PLHIV (18 children, 36 maternal deaths, and 110 adults). HIV antibody levels and HIV RNA viral loads were determined from postmortem serum samples.

**Results:**

Tuberculosis (22.7%), toxoplasmosis (13.9%), bacterial infections (13.9%), and cryptococcosis (10.9%) were the leading causes of death in adults. In maternal deaths, tuberculosis (13.9%), bacterial infections (13.9%), cryptococcosis (11.1%), and cerebral malaria (8.3%) were the most frequent infections, whereas viral infections, particularly cytomegalovirus (38.9%), bacterial infections (27.8%), pneumocystosis (11.1%), and HIV-associated malignant neoplasms (11.1%) were the leading cause among children. Agreement between the MITS and the complete autopsy was 100% in children, 91% in adults, and 78% in maternal deaths. The MITS correctly identified the microorganism causing death in 89% of cases.

**Conclusions:**

Postmortem studies provide highly granular data on the causes of death in PLHIV. The inaccuracy of clinical diagnosis may play a significant role in the high mortality rates observed among PLHIV in LMICs. MITS might be helpful in monitoring the causes of death in PLHIV and in highlighting the gaps in the management of the infections.

The scale-up of antiretroviral therapy (ART) has resulted in a decline in AIDS-associated mortality rates globally, from a peak of 1.9 million estimated deaths in 2004 to about 690 000 in 2019 [1]. Despite this achievement, reaching the United Nations General Assembly 2020 milestone of <500 000 annual AIDS deaths remains elusive. Of the 38 million people living with human immunodeficiency virus (PLHIV) globally, about 12 million (32%) do not receive ART, most of them in low- and middle-income countries (LMICs) [2, 3]. Moreover, about 30% of new HIV infections are diagnosed at advanced stage, which is a strong predictor of morbidity and mortality risk [4, 5].

Increasing knowledge on the causes of AIDS deaths is key to ending AIDS. However, most of the available knowledge on HIV-related mortality rates in LMICs is not based on postmortem data but on indirect estimations [6–8], including clinical records and verbal autopsies, with important limitations [9, 10]. A 2015 meta-analysis on the contribution of tuberculosis to AIDS-related mortality rates, including 36 studies and >3200 autopsies, identified tuberculosis as the cause of 40% of in-hospital AIDS-related deaths in LMICs [11]. Cryptococcal meningitis is considered to be responsible for 15% of AIDS-related deaths globally [12], but lack of postmortem studies seriously hampers this estimation. Beyond tuberculosis and cryptococcosis, the knowledge on the relative contribution of other causes, such as severe bacterial infections, toxoplasmosis and *Pneumocystis* pneumonia in adults, and severe viral and bacterial infections, *Pneumocystis* pneumonia, diarrheal disease, and malnutrition in children, is scarce [6–8].

To improve AIDS mortality data globally, postmortem studies need to be expanded to regions with high HIV burden. The complete autopsy is the reference standard to determine the cause of death, but it remains unpractical in resource-limited settings, owing to lack of resources and trained personnel, limited acceptability of the procedure, and the high number of deaths occurring outside the health facilities. A minimally invasive tissue sampling (MITS) approach, involving the sampling of key organs and fluids for histological and microbiological analysis, has been developed to be used in LMICs [13]. This method has high concordance against complete autopsies, particularly for infectious diseases [14], can be performed by less qualified staff, has high acceptability [15], and can be successfully implemented including outside health facilities.

MITS has been validated in a series of in-hospital stillbirth, neonatal, pediatric, adult, and maternal deaths in Mozambique and Brazil [13, 16–19]. It has a potential role in identifying implementation gaps and guiding efforts to reduce AIDS-related mortality rates in LMICs. The current study aimed to (1) provide high-quality postmortem data on causes of death in PLHIV; (2) assess the role of clinical misdiagnosis in these deaths; and (3) evaluate the accuracy of MITS in determining the cause of death.

## METHODS

### Study Setting

CaDMIA is an international observational study conducted in two sites: the Maputo Central Hospital in Maputo, Mozambique, a 1500-bed government-funded quaternary hospital, and the Fundação de Medicina Tropical Doutor Heitor Vieira Dourado in Manaus, Brazil, a 143-bed government-funded hospital and referral infectious diseases hospital for the Amazon state. In-hospital deaths of all ages between November 2013 and March 2015 were eligible if they fulfilled these criteria: (1) a complete autopsy requested by the clinician as part of the medical evaluation of the patient and (2) informed consent to perform the postmortem examinations, given by the relatives. In this substudy, we included all HIV-positive patients tested either during admission or in the postmortem evaluation.

### Postmortem Procedures

The detailed MITS protocol has been reported elsewhere [14, 20]. Briefly, the procedure included disinfection of the body surface, followed by the collection of blood and cerebrospinal fluid (CSF) and puncture of the liver, lungs, and central nervous system (CNS) for microbiological and pathological analysis, using biopsy needles. A second pathologist not involved in the MITS performed a complete autopsy, following a standardized protocol. The histological evaluation included staining with hematoxylin-eosin of all MITS and complete autopsy samples and additional histochemical and/or immunohistochemical staining whenever needed.

The detection of antibodies against HIV-1/2 and quantification of the HIV RNA viral load were assessed in the blood obtained during the MITS. We performed a universal screening, including hepatitis B and C viruses serology; bacterial and fungal cultures of blood, CSF, liver, lungs, and CNS; and polymerase chain reaction PCR for *Plasmodium falciparum*. In addition, real-time PCR was routinely applied in CSF and CNS samples to screen for *Toxoplasma gondii, Mycobacterium tuberculosis,* and *Cryptococcus* spp., and in lung samples for *Pneumocystis jirovecii, Cryptococcus* spp., and *M. tuberculosis*. In all lung samples from children, we performed multiplexed PCR analyses for common respiratory viruses and bacteria. Other microorganisms were also tested, depending on the histological features observed in the autopsy samples [14, 20].

### Determination of the Cause of Death

A panel composed of a pathologist, a microbiologist, and an infectious diseases specialist evaluated all the MITS data blinded to any clinical data and assigned the MITS diagnosis. After a 3–6-month washout period, the same panel evaluated the data from the complete autopsy and the clinical records and assigned the final cause of death. All conditions directly leading to death, any underlying conditions, as well as other significant conditions possibly contributing to death, were independently coded following the *International Classification of Diseases and Related Health Problems, Tenth Revision* (*ICD-10*) [21].

The main causes of death and contributing conditions were classified into four major groups: infectious diseases, cancers, other diseases (cardiovascular, gastrointestinal, kidney, lung diseases, and in maternal deaths, obstetric conditions), and nonconclusive. When more than one severe pathological and/or microbiological diagnosis was identified, the disease most likely causing death was considered the final cause of death.

### Assessment of Clinical-Pathological Discrepancies

Diagnostic discrepancies were classified following the classification of Goldman et al [22], modified by Battle et al [23]. Major discrepancies (classes I and II) were those involving the cause of death or the underlying condition. In class I, the knowledge of the diagnosis before death, would have led to changes in the management that could have prolonged the survival or cured the patient, while in class II the survival would not have been modified. Minor discrepancies (classes III and IV) involved other associated conditions. Correctly diagnosed cases were classified as class V, and nonclassifiable cases as class VI. Each case was assessed by 2 independent raters, with discrepancies solved by a third rater, following a procedure described elsewhere [9].

### Statistical Analysis

Comparisons between groups were made using Student *t* or Mann-Whitney *U* tests for continuous variables and χ ^2^ or Fisher exact tests for categorical variables, as appropriate. The methods for assessing the level of agreement between the MITS and the complete autopsy diagnoses are described elsewhere [16–18]. Briefly, the agreement between both methods was assessed by *ICD-10* code comparison, which classifies diagnoses into chapters, blocks, and 3-character categories [21]. The agreement was classified as perfect (identical *ICD-10* codes in the chapter, block, and 3-character category), moderate (discrepancy in the 3-character category), low (discrepancy in the block and 3-character category), and none (discrepancy in the chapter).

### Ethical Considerations

The study received ethical approval from the National Health Bioethics Committee of Mozambique (reference 342/CNBS/13), the National Committee for Ethics in Research of Brazil (reference 1.074.304), and the Clinical Research Ethics Committee of the Hospital Clinic of Barcelona (file 2013/8677).

## RESULTS

### Baseline and HIV Characteristics

We included 164 deceased PLHIV, 127 from Mozambique and 37 from Brazil. A summary of the baseline demographic features, as well as the general characteristics of the HIV infection of the patients included in this series are presented in Table 1. All patients from Mozambique were black. In Brazil, 43%, 16%, and 41% were white, indigenous American, and ethnically mixed, respectively.

**Table 1.** Baseline and Human Immunodeficiency Virus Characteristics by Site

	Patients, No. (%)^a^		
Characteristic	Mozambique (n = 127)	Brazil (n = 37)	*P* Value^b^
Group			
Adults	73 (57.5)	37 (100)	<.001
Maternal deaths	36 (28.3)	0 (0)	
Children	18 (14.2)^c^	0 (0)	
Sex			
Male	48 (37.8)	26 (70.3)	<.001
Female	79 (62.2)	11 (29.7)	
Age, mean (SD), y	31 (15)	38 (11)	.009^d^
Residence living in urban setting	96 (75.6)	36 (97.3)	.002
Clinical diagnosis of HIV infection			
Diagnosed before admission	90 (70.9)	27 (73.0)	.09
Diagnosed during admission	15 (11.8)	8 (21.6)	
Not diagnosed	22 (19.2)	2 (5.4)	
CD4 cell count and viral load available	34 (26.8)	28 (75.7)	<.001
Receipt of ART	66 (52.0)	12 (32.4)	.04
Receipt of prophylactic cotrimoxazole	53 (41.7)	0 (0)	<.001

Abbreviations: ART, antiretroviral therapy; HIV, human immunodeficiency virus; SD, standard deviation.

^a^Data represent no. (%) of patients unless otherwise specified.

^b^P values calculated with Fisher exact test, unless otherwise notes.

^c^Including 1 neonate.

^d^Calculated with Student *t* test.

Most patients had HIV infection diagnosed before death (83% from Mozambique and 95% from Brazil). The median last CD4 cell count before death was 66/μL, and the median HIV RNA viral load was 4.1 log_10_ copies/mL. Forty-eight percent of patients had been started on ART (52% in Mozambique and 32% in Brazil), and the median time on ART at death was 5 months. Forty-two percent of participants had received prophylactic cotrimoxazole in Mozambique, but none in Brazil. None of the patients had received tuberculosis preventive therapy.

### Main Causes of Death

The causes of death identified by the complete autopsy disaggregated by age group are shown in Table 2. Overall, tuberculosis was identified as the main cause of death in 18.3% of cases, cryptococcosis in 9.8%, toxoplasmosis in 9.1%, and *Pneumocystis* pneumonia in 3.0%. Invasive bacterial infections caused 15.2% of deaths (25 of 164), with the predominant bacteria being *Streptococcus pneumoniae* (7 of 25), other gram-positive cocci (3 of 25), gram-negative bacteria (14 of 25), and *Mycoplasma pneumoniae* (1 of 25). Viral infections caused 9.1% of all deaths (15 of 164), mostly due to cytomegalovirus (12 of 15). Cancers were the main cause of death in 10.4% of cases (17 of 164), including malignant lymphoma/leukemia (9 of 17), Kaposi sarcoma (4 of 17), carcinoma of the uterine cervix (2 of 17), breast cancer (1 of 17), and extrahepatic cholangiocarcinoma (1 of 17).

**Table 2.** Causes of Death Identified by Complete Autopsy, by Age Group

	Deaths, No. (%)			
Cause of Death	Adults (n = 110)	Maternal Deaths (n = 36)	Children^a^ (n = 18)	*P* Value
Malignant tumors	14 (12.7)	1 (2.8)	2 (11.1)	.23
Other diseases	4 (3.6)	10^b^ (27.8)	0 (0)	<.001
Nonconclusive	0 (0)	1 (2.8)	0 (0)	.33
Infections	92 (83.6)	24 (66.7)	16 (88.9)	.07
Specific infections				
Tuberculosis	25 (22.7)	5 (13.9)	0 (0)	.04
Cryptococcosis	12 (10.9)	4 (11.1)	0 (0)	.39
Histoplasmosis	6 (5.4)	NA^c^	NA^c^	
Pneumocystosis	3 (2.7)	0 (0)	2 (11.1)	.12
Other fungal disease	1 (0.9)	0 (0)	0 (0)	>.99
Toxoplasmosis	15 (13.6)	0 (0)	0 (0)	.02
Cerebral malaria	0 (0)	3 (8.3)	0 (0)	.02
Bacterial infection	15 (13.6)	5 (13.9)	5 (27.8)	.27
Viral infection	8 (7.3)	0 (0)	7^a^ (38.9)	<.001
Infection, no microorganism identified	7 (6.4)	8 (22.2)	2 (11.1)	.02

Abbreviation: NA, not applicable.

^a^Including 1 neonate.

^b^For the maternal deaths, obstetric conditions were categorized as other diseases.

^c^No maternal deaths or children were included in Brazil, where all deaths related to histoplasmosis occurred.

**Figure 1.**
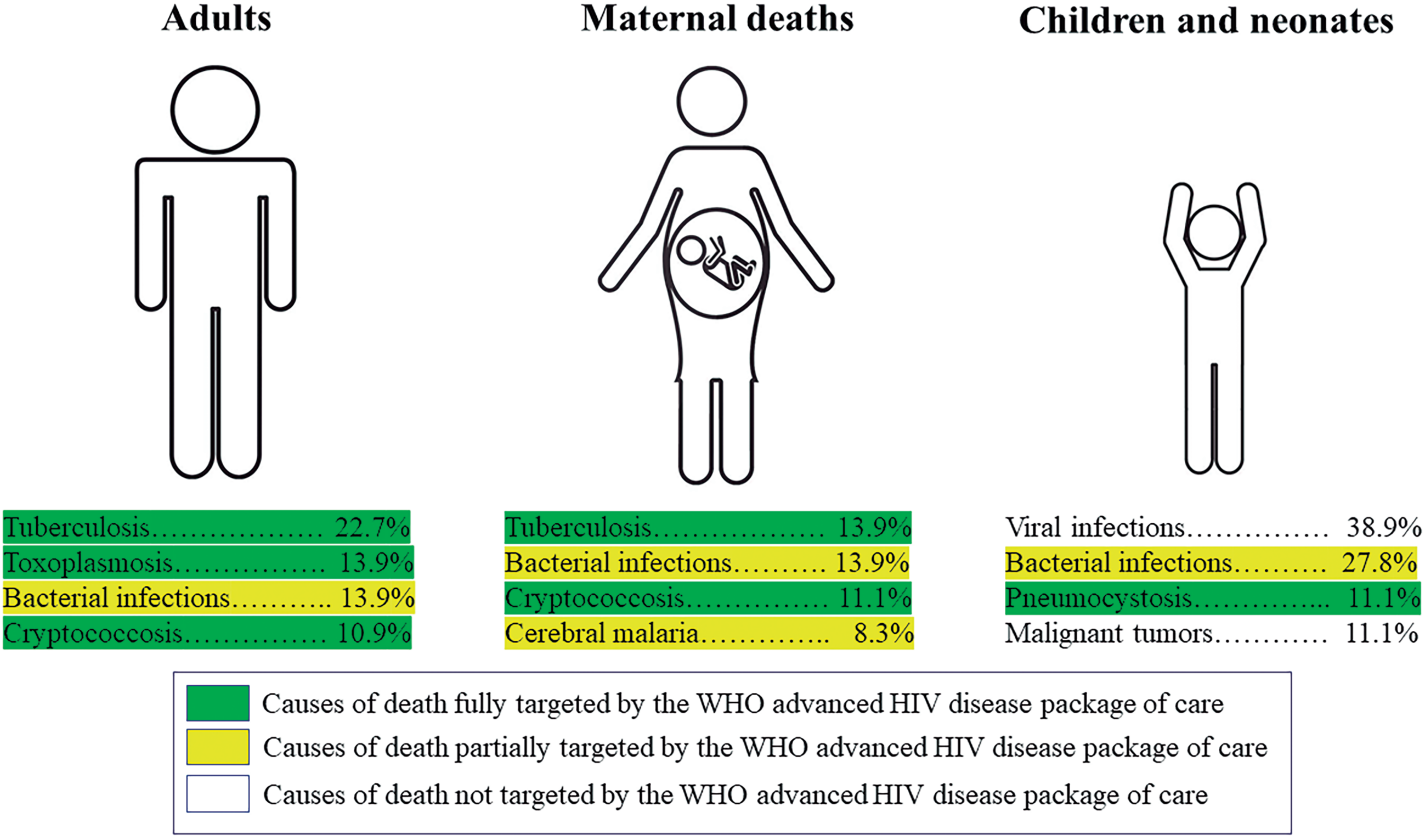
Major causes of death in adults, maternal deaths, and children and neonates. The World Health Organization (WHO) package of care for advanced human immunodeficiency virus (HIV) disease is a package of interventions including tuberculosis and cryptococcosis screening, treatment and/or prophylaxis for major opportunistic infections (cotrimoxazole, tuberculosis preventive treatment, fluconazole preemptive therapy), rapid antiretroviral therapy initiation and intensified adherence support interventions recommended to everyone presenting with advanced HIV disease (strong recommendation, moderate-quality evidence).

Tuberculosis was the main cause of death among adults, followed by toxoplasmosis, bacterial infections, and cryptococcosis. Tuberculosis and bacterial infections, followed by cryptococcosis and cerebral malaria, were the leading infections causing maternal death. Among children, viruses, particularly cytomegalovirus, bacterial infections, mainly *S. pneumoniae,* and *Pneumocystis* pneumonia, were the most common causes of death. Most deaths were caused by preventable diseases (Figure 1). The specific cause of death of each case is shown in Supplementary Table 1.

Table 3 summarizes the main causes of death in the 110 nonpregnant adults disaggregated by site. Significant differences were observed for bacterial infections, more frequently identified in Mozambique, and histoplasmosis, identified as the cause of death in 6 (16.2%) of the patients in Brazil and in none in Mozambique.

**Table 3.** Main Causes of Death Identified by Complete Autopsy in Adult Patients (Excluding Maternal Deaths), by Site

	Deaths, No. (%)		*P* Value
Cause of Death	Mozambique (n = 73)	Brazil (n = 37)	
Malignant tumors	10 (13.7)	4 (10.8)	.41
Other diseases	4 (5.5)	0 (0)	
Nonconclusive	0 (0)	0 (0)	
Infections	59 (80.8)	33 (89.2)	
Specific infections			
Tuberculosis	18 (24.7)	7 (18.9)	.82
Cryptococcosis	7 (9.6)	5 (13.5)	.53
Histoplasmosis	0 (0)	6 (16.2)	.004
Pneumocystosis	2 (2.7)	1 (2.7)	>.99
Other fungal disease	1 (1.4)	0 (0)	>.99
Toxoplasmosis	9 (12.3)	6 (16.2)	.57
Bacterial infection	14 (19.2)	1 (2.7)	.02
Viral infection	4 (5.5)	4 (10.8)	.44
Infection with no microorganism identified	4 (5.5)	3 (8.1)	.69

### Other Diseases Identified

Other infectious diseases contributing to death were identified in 42 patients (25.6%), including 30 adults (26.8%) and 12 maternal deaths (33.3%). In 10 cases, these additional infections were multiple. These included cytomegalovirus (17 cases), bacterial infections (11 cases), toxoplasmosis (10 cases), tuberculosis (8 cases), histoplasmosis (8 cases), and *Pneumocystis* pneumonia (5 cases). When including these additional infections contributing to death, toxoplasmosis was identified in 9 of 73 adults (15.3%) from Mozambique and in 16 of 37 patients (43%) from Brazil (*P* < .001). Other associated noninfectious diseases were identified in 18 patients (11%), including chronic hepatitis or cirrhosis (all caused by hepatitis B virus), and cardiovascular diseases (7 cases each).

### Clinical-Pathological Discrepancies

A major clinical-pathological diagnostic discrepancy was detected in 89 deaths (54.3%), including 83 class I and 6 class II errors. A minor diagnostic discrepancy (class III or IV) was identified in 19 cases (11.6%; 13 class III and 6 class IV errors). Complete agreement (class V) was seen in 55 cases (33.5%). Table 4 shows distribution of the clinical errors in each age group for the overall diagnoses, and Table 5 the number and percentage of major errors identified for each specific infection.

**Table 4.** Distribution of Clinical Errors in Each Age Group for the Overall Diagnoses

	Clinical Errors, No. (%)			
Class of Error	Children^a^ (n = 18)	Maternal Deaths (n = 36)	Adults (n = 110)	Total (n = 164)
Class I (major)	7 (38.9)	12 (33.3)	64 (58.3)	83 (50.6)
Class II (major)	1 (5.6)	3 (8.3)	2 (1.8)	6 (3.7)
Class III (minor)	3 (16.6)	9 (25.0)	1 (0.9)	13 (7.9)
Class IV (minor)	0 (0)	1 (2.8)	5 (4.5)	6 (3.7)
Class V (no error)	7 (38.9)	10 (27.8)	38 (34.5)	55 (33.5)
Class VI (no diagnosis)	0 (0)	1 (2.8)	0 (0)	1 (0.6)

^a^Including 1 neonate.

**Table 5.** Major Diagnostic Errors by Cause of Death and Specific Infection

Diagnosis	Cases Diagnosed at Autopsy, No.	Major Clinical Errors, No. (%)
Cause of death		
Malignant tumors	17	7 (41.2)
Other diseases	14	2 (14.3)
Nonconclusive	1	0 (0)
Infections	132	80 (60.5)
Specific infections		
Tuberculosis	30	14 (46.7)
Cryptococcosis	16	9 (56.2)
Histoplasmosis	6	4 (66.7)
Pneumocystosis	5	3 (60.0)
Cerebral malaria	3	1 (33.3)
Other fungal disease	1	1 (100)
Toxoplasmosis	15	13 (86.7)
Bacterial infection	25	16 (64.0)
Viral infection	15	11 (73.3)

### Agreement Between the MITS and the Complete Autopsy

The level of agreement between MITS and complete autopsy was perfect in 78.0% of cases (128 of 164), moderate in 2.4% (4 of 164), low in 3.0% (5 of 164), and none in 16% (27 of 164). The agreement was highest for infectious diseases (88.4% [107 of 121] overall, 100% [16 of 16] among children, and 98.8% [85 of 86] among adults). The MITS correctly identified the microorganism causing death in 103 of 116 cases (88.8%). Table 6 shows the agreement between the MITS and the complete autopsy for the different diagnoses. The diagnostic agreement between MITS and complete autopsy was significantly higher in HIV-positive than in HIV-negative patients (78% vs 58%; *P* = .01; data not shown).

**Table 6.** Agreement Between Minimally Invasive Tissue Sampling and the Complete Autopsy for Different Diagnoses

Diagnosis	Cases, No.	Correctly Diagnosed by MITS (Perfect or Almost Perfect Agreement), No.(%)
Cause of death		
Infections	132	111 (84.1)
Malignant tumors	17	14 (82.4)
Other diseases	14	4 (28.5)
Nonconclusive	1	1 (100)
Specific infection		
Tuberculosis	30	28 (93.3)
Cryptococcosis	16	14 (87.5)
Histoplasmosis	6	6 (100)
Pneumocystosis	5	5 (100)
Cerebral malaria	3	3 (100)
Other fungal disease	1	1 (100)
Toxoplasmosis	15	12 (80.0)
Bacterial infection	25	22 (88.0)
Viral infection	15	13 (86.6)
Infection, no microorganism identified	16	7 (43.7)

Abbreviation: MITS, minimally invasive tissue sampling.

## Discussion

This is one of the few autopsy-based studies analyzing the causes of death among PLHIV in LMICs. The results provide an accurate identification of the fatal HIV-associated opportunistic infections, and they reflect marked age differences and geographic variations. The study also shows an excellent agreement between the MITS and the complete autopsy. Finally, it identifies a high proportion of clinical diagnostic errors, providing insight on existing gaps in the management of opportunistic infections. Remarkably, most deaths were due to preventable causes.

The study population characteristics reflect those of PLHIV presenting with advanced HIV disease (AHD) in sub-Saharan Africa and Latin America. Interestingly, 86% of patients had HIV infection diagnosed before death, but only 34% in Brazil and 52% in Mozambique had started ART. Testing people earlier, ensuring effective linkage and timely ART initiation, and maximizing ART adherence and long-term retention in HIV care are essential steps in reducing mortality rates.

Tuberculosis was the leading cause of death in adults [24]. Remarkably, tuberculosis had not been suspected in 66% of the cases, and no patient had received recommended tuberculosis-preventive treatment. Although such treatment has been shown to reduce mortality rates in PLHIV [25], only one-third of LMICs are currently implementing it [26]. Moreover, the World Health Organization (WHO) recommends the tuberculosis lipoarabinomannan antigen, shown to increase diagnoses and reduce mortality rates in PLHIV with CD4 cell counts <100/μL [27]. Our results highlight the need of scaling up these life-saving interventions.

Cryptococcosis caused 10.9% and 11.1% of adult and maternal deaths, respectively, with more than half of the patients receiving ART. Diagnostic facilities, access to optimal antifungal medications, and intensive hospital-based treatments are limited in LMICs [12]. The development of a point-of-care lateral flow assay for cryptococcal antigen, allowing treatment of those with cryptococcal antigenemia with high-dose fluconazole before meningitis onset, has shown to reduce mortality rates [28, 29]. This cost-effective and life-saving strategy is recommended by the WHO [30], but its uptake remains minimal in LMICs.

Toxoplasmosis was the second most frequent cause of death in adults (13.9%). Worrisomely, it was clinically misdiagnosed in 86.7% of cases. The proportion of PLHIV receiving cotrimoxazole preventive treatment (CPT) was 50% in Mozambique and 0% in Brazil. Whereas, in the absence of appropriate prophylaxis, toxoplasmosis is the most common opportunistic infection of the CNS in PLHIV [31], the risk in patients receiving CPT approaches 0% [32]. The proportion of patients from Mozambique in whom toxoplasmosis developed despite CPT suggests deficiencies in its implementation. It is fundamental that this life-saving and cost-effective intervention is consistently implemented in all LMICs.

Histoplasmosis caused 16% of deaths in Brazil. Despite being more prevalent than tuberculosis in most Latin American countries, it remains underdiagnosed and often misdiagnosed as tuberculosis [33]. In our study, none of the histoplasmosis cases had been identified and treated before death. This stresses the need for increased awareness, early diagnosis, and availability of rapid point-of-care tests in endemic areas [33].

Viral infections, mostly cytomegalovirus, were the most frequent cause of death among children. Cytomegalovirus treatment is expensive and hardly available in LMICs [34]. Prevention involves cytomegalovirus-seronegative blood for preterm infants and treatment of maternal breast milk through freezing or pasteurization, not available in LMICs. Thus, the prevention of cytomegalovirus infection and mother-to-child transmission relies on universal HIV testing among pregnant women plus immediate ART for all children <5 years old, essential steps in reducing pediatric AIDS-related mortality rates.

Invasive bacterial infections caused more than a quarter of deaths in children and 14% of adult and maternal deaths, a significant proportion by *S. pneumoniae.* The WHO recommends including pneumococcal conjugate vaccines in childhood immunization programs worldwide [35], and coverage is high in Mozambique and Brazil. Among adult PLHIV, vaccination has been recommended since the mid-1990s [36]. However, despite evidence of significant efficacy and immunogenicity of the 7-valent conjugate vaccine among adult PLHIV from Malawi [37], pneumococcal immunization is not yet available for PLHIV in Africa.

*Pneumocystis* pneumonia was particularly relevant among children. Once established, the mortality rate in PLHIV ranges from 5% to 40% if *Pneumocystis* pneumonia is treated and approaches 100% if it is untreated. Early identification of HIV-exposed infants and timely initiation of cotrimoxazole prophylaxis and ART is essential [38].

Malignant neoplasms were the main cause of death in 10% of cases, including Kaposi sarcoma, hematologic cancers, and cervical cancer, all AIDS-defining cancers arising in PLHIV with AHD. A recent population-based cancer registry found that 44% of cancers in Maputo are caused by infectious agents [39]. This underscores the importance of widespread HIV testing and rapid ART initiation.

The exceptionally high level of agreement between the MITS and the complete autopsy for infectious diseases highlights the potential of the MITS to reliably ascertain the causes of death among PLHIV. The MITS has been validated in a multisite study and has shown high acceptability by the population in LMICs [15]. Our results emphasize the potential use to identify program gaps in different regions and urgently address the AIDS-related mortality risk.

Finally, the WHO has issued specific guidelines for managing AHD, recommending offering a package of care to all PLHIV presenting with AHD, including screening for opportunistic infections through point-of-care testing (cryptococcal antigen lateral flow assay and tuberculosis lipoarabinomannan antigen), preemptive/prophylactic treatment of opportunistic infections, and accelerated ART [40]. In our study, overall, 40% of all deaths were caused by tuberculosis, cryptococcosis, toxoplasmosis, and *Pneumocystis* pneumonia, diseases directly targeted by this package of care. Another 19% of patients died of bacterial infections partially targeted by cotrimoxazole prophylaxis (Figure 1). Thus, our results support the urgent implementation of these guidelines, which could have a significant impact on AIDS-related mortality rates in LMICs.

In conclusion, our study provides an accurate description of the opportunistic infection causing death in PLHIV and highlights gaps in the management of these infections that hamper clinic outcomes. The study identifies a high level of clinical-pathological discrepancies and confirms the excellent agreement between the MITS and the complete autopsy among PLHIV. These findings encourage the widespread implementation of recommended guidelines and the use of the MITS to monitor the causes of death in PLHIV and address the implementation gaps to reduce the still unacceptably high AIDS-related mortality rates in LMICs.

## Supplementary Data

Supplementary materials are available at *Clinical Infectious Diseases* online. Consisting of data provided by the authors to benefit the reader, the posted materials are not copyedited and are the sole responsibility of the authors, so questions or comments should be addressed to the corresponding author.

ciab789_suppl_Supplementary_Table_1

## Notes

***Acknowledgments.*** We thank the families of the deceased patients included in this study. We are grateful to all the members of the Department of Pathology of the Maputo Central Hospital (MCH), whose support made this study possible and also to the staff of the Centro de Investigação em Saúde de Manhiça for their logistic support. We specifically thank Bento Nhancale for his invaluable support of the study.

***Supplement sponsorship.*** This supplement is sponsored by MITS Surveillance Alliance Secretariat, led by RTI International, with funding from the Bill and Melinda Gates Foundation.

***Disclaimer.*** The funders had no role in study design, data collection and analysis, decision to publish, or preparation of the manuscript.

***Financial support.*** This work was supported by the Bill & Melinda Gates Foundation (grants OPP1067522 and OPP1128001), the Spanish Instituto de Salud Carlos III (grant PI12/00757)), the Government of Mozambique and the Spanish Agency for International Development (support to the Centro de Investigação em Saúde de Manhiça), the Spanish Ministry of Science and Innovation through the Centro de Excelencia Severo Ochoa 2019–2023 program (grant CEX2018-000806-S to ISGlobal), and the Generalitat de Catalunya through the CERCA Program (support to ISGlobal).

***Potential conflicts of interest.*** All authors: No reported conflicts. All authors have submitted the ICMJE Form for Disclosure of Potential Conflicts of Interest. Conflicts that the editors consider relevant to the content of the manuscript have been disclosed.
