# Supplementary material for: Minimally Invasive Tissue Sampling: A Tool to Guide Efforts to Reduce AIDS-Related Mortality in Resource-Limited Settings
Source: Clin Infect Dis. 2021 Dec 15;73(Suppl 5):S343–50. doi: 10.1093/cid/ciab789 (PMC8672756; doi:10.1093/cid/ciab789)
Supplement: ciab789_suppl_Supplementary_Table_1 [file ciab789_suppl_supplementary_table_1.docx]

**Supplementary Table 1.** Age group, age, sex and cause of death of each case.

| **Case** | **Country** | **Age group** | **Age** | **Sex** | **HIV** | **Cause of death** | **Associated conditions** |
| --- | --- | --- | --- | --- | --- | --- | --- |
| 1 | Mozambique | Adult | 28 | Female | Positive | Disseminated Kaposi’s sarcoma |  |
| 2 | Mozambique | Adult | 35 | Female | Positive | Pulmonary Pneumocystosis | Cirrhosis |
| 3 | Mozambique | Adult | 35 | Female | Positive | Disseminated Toxoplasmosis |  |
| 4 | Mozambique | Adult | 27 | Female | Positive | Pneumonia (Acinetobacter) |  |
| 5 | Mozambique | Adult | 41 | Male | Positive | Meningitis (S. pneumoniae) |  |
| 6 | Mozambique | Adult | 33 | Male | Positive | Encephalitis (CMV) |  |
| 7 | Mozambique | Adult | 17 | Female | Positive | Meningitis (M. Tuberculosis) |  |
| 8 | Mozambique | Adult | 43 | Male | Positive | Pulmonary Pneumocystosis |  |
| 9 | Mozambique | Adult | 53 | Female | Positive | Disseminated Large B cell lymphoma |  |
| 10 | Mozambique | Adult | 36 | Female | Positive | Carcinoma, uterine cervix |  |
| 11 | Mozambique | Adult | 35 | Male | Positive | Pneumonia (Adenovirus) |  |
| 12 | Mozambique | Adult | 29 | Female | Positive | Milliary Tuberculosis |  |
| 13 | Mozambique | Adult | 57 | Female | Positive | Carcinoma, uterine cervix |  |
| 14 | Mozambique | Adult | 45 | Female | Positive | Milliary Tuberculosis |  |
| 15 | Mozambique | Adult | 29 | Female | Positive | Disseminated Cryptococcosis |  |
| 16 | Mozambique | Adult | 32 | Male | Positive | Sepsis (S. dysgalactiae) |  |
| 17 | Mozambique | Adult | 30 | Female | Positive | Cerebral Toxoplasmosis |  |
| 18 | Mozambique | Adult | 38 | Male | Positive | Pneumonia (K. pneumoniae) |  |
| 19 | Mozambique | Adult | 32 | Male | Positive | Disseminated Toxoplasmosis | CMV, Prevotella, S. pneumoniae, HSV-1 |
| 20 | Mozambique | Adult | 39 | Male | Positive | Large B cell lymphoma |  |
| 21 | Mozambique | Adult | 37 | Male | Positive | Sepsis (No microorganism) | Cirrhosis |
| 22 | Mozambique | Adult | 60 | Female | Positive | Acute myocardial infarction | Hypertension |
| 23 | Mozambique | Adult | 29 | Female | Positive | Pneumonia (No microorganism) | Chronic hepatitis |
| 24 | Mozambique | Adult | 32 | Female | Positive | Disseminated Toxoplasmosis |  |
| 25 | Mozambique | Adult | 57 | Female | Positive | Sepsis (E. coli) | Diabetes Mellitus 2 |
| 26 | Mozambique | Adult | 34 | Male | Positive | Disseminated Cryptococcosis |  |
| 27 | Mozambique | Adult | 44 | Female | Positive | Pneumonia (E. coli) |  |
| 28 | Mozambique | Adult | 50 | Male | Positive | Miliary tuberculosis |  |
| 29 | Mozambique | Adult | 36 | Male | Positive | Disseminated Cryptococcosis |  |
| 30 | Mozambique | Adult | 36 | Male | Positive | Pneumonia (K. pneumoniae) |  |
| 31 | Mozambique | Adult | 50 | Male | Positive | Meningitis (S. pneumoniae) | Chronic hepatitis |
| 32 | Mozambique | Adult | 55 | Female | Positive | Disseminated infection (HHV-1) |  |
| 33 | Mozambique | Adult | 40 | Female | Positive | Pulmonary CMV | HSV-1 |
| 34 | Mozambique | Adult | 34 | Male | Positive | Sepsis (E. coli) |  |
| 35 | Mozambique | Adult | 32 | Female | Positive | Gastroenteritis |  |
| 36 | Mozambique | Adult | 15 | Male | Positive | Sepsis (C. glabrata) |  |
| 37 | Mozambique | Adult | 44 | Male | Positive | Disseminated Cryptococcosis | Chronic viral hepatitis (HBV) |
| 38 | Mozambique | Adult | 34 | Female | Positive | Pulmonary Tuberculosis |  |
| 39 | Mozambique | Adult | 46 | Male | Positive | Acute myeloid leukemia |  |
| 40 | Mozambique | Adult | 55 | Male | Positive | Sepsis (S. typhi) |  |
| 41 | Mozambique | Adult | 37 | Female | Positive | Dilated cardiomyopathy | Hypertensive heart and renal disease |
| 42 | Mozambique | Adult | 43 | Male | Positive | Disseminated Cryptococcosis |  |
| 43 | Mozambique | Adult | 54 | Male | Positive | Sepsis (E. coli) |  |
| 44 | Mozambique | Adult | 25 | Male | Positive | Disseminated Cryptococcosis |  |
| 45 | Mozambique | Adult | 40 | Female | Positive | Disseminated Toxoplasmosis | Pneumonia (Pneumocystis) |
| 46 | Mozambique | Adult | 59 | Male | Positive | Pulmonary Toxoplasmosis | Cardiopathy |
| 47 | Mozambique | Adult | 36 | Female | Positive | Pneumonia (Legionella) |  |
| 48 | Mozambique | Adult | 35 | Female | Positive | Cerebral Cryptococcosis |  |
| 49 | Mozambique | Adult | 46 | Female | Positive | Miliary tuberculosis |  |
| 50 | Mozambique | Adult | 45 | Female | Positive | Cerebral hemorrhage | Hypertension |
| 51 | Mozambique | Adult | 32 | Female | Positive | Miliary tuberculosis |  |
| 52 | Mozambique | Adult | 23 | Female | Positive | Cerebral Toxoplasmosis |  |
| 53 | Mozambique | Adult | 58 | Female | Positive | Disseminated Undifferentiated neoplasm |  |
| 54 | Mozambique | Adult | 33 | Female | Positive | Miliary tuberculosis | Cirrhosis |
| 55 | Mozambique | Adult | 34 | Male | Positive | Disseminated Kaposi’s sarcoma |  |
| 56 | Mozambique | Adult | 40 | Male | Positive | Pneumonia (E. coli) | Papillary renal cell carcinoma |
| 57 | Mozambique | Adult | 56 | Female | Positive | Cerebral hemorrhage | Hypertension |
| 58 | Mozambique | Adult | 39 | Female | Positive | Disseminated Large B cell lymphoma |  |
| 59 | Mozambique | Adult | 30 | Female | Positive | Hepatocellular carcinoma | HBV |
| 60 | Mozambique | Adult | 38 | Male | Positive | Miliary tuberculosis |  |
| 61 | Mozambique | Adult | 46 | Male | Positive | Miliary tuberculosis |  |
| 62 | Mozambique | Adult | 52 | Male | Positive | No microorganism |  |
| 63 | Mozambique | Adult | 46 | Female | Positive | Miliary tuberculosis |  |
| 64 | Mozambique | Adult | 38 | Male | Positive | Miliary tuberculosis |  |
| 65 | Mozambique | Adult | 33 | Male | Positive | Miliary tuberculosis |  |
| 66 | Mozambique | Adult | 49 | Male | Positive | Pneumonia (Pseudomonas) |  |
| 67 | Mozambique | Adult | 29 | Female | Positive | Disseminated Toxoplasmosis |  |
| 68 | Mozambique | Adult | 38 | Female | Positive | Disseminated Toxoplasmosis |  |
| 69 | Mozambique | Adult | 35 | Male | Positive | Miliary tuberculosis |  |
| 70 | Mozambique | Adult | 47 | Male | Positive | Miliary tuberculosis |  |
| 71 | Mozambique | Adult | 32 | Male | Positive | Miliary tuberculosis |  |
| 72 | Mozambique | Adult | 36 | Male | Positive | Miliary tuberculosis |  |
| 73 | Mozambique | Adult | 26 | Male | Positive | Miliary tuberculosis |  |
| 74 | Brazil | Adult | 31 | Female | Positive | Miliary tuberculosis | Steatohepatitis |
| 75 | Brazil | Adult | 38 | Male | Positive | Disseminated Toxoplasmosis | CMV |
| 76 | Brazil | Adult | 29 | Male | Positive | Miliary tuberculosis | Toxoplasma |
| 77 | Brazil | Adult | 28 | Male | Positive | Disseminated Cryptococcosis | Tuberculosis |
| 78 | Brazil | Adult | 32 | Male | Positive | Disseminated Cryptococcosis | K. pneumoniae |
| 79 | Brazil | Adult | 52 | Male | Positive | Miliary tuberculosis | Toxoplasma |
| 80 | Brazil | Adult | 33 | Female | Positive | Disseminated Large B cell lymphoma | Tuberculosis |
| 81 | Brazil | Adult | 31 | Male | Positive | Hemorrhagic disease | S. pneumoniae |
| 82 | Brazil | Adult | 25 | Male | Positive | Disseminated Kaposi's sarcoma |  |
| 83 | Brazil | Adult | 39 | Female | Positive | Breast cancer |  |
| 84 | Brazil | Adult | 55 | Female | Positive | Bile duct carcinoma |  |
| 85 | Brazil | Adult | 48 | Male | Positive | Disseminated histoplasmosis | E. coli, CMV, toxoplasma |
| 86 | Brazil | Adult | 25 | Female | Positive | Miliary tuberculosis |  |
| 87 | Brazil | Adult | 32 | Male | Positive | Disseminated histoplasmosis | Toxoplasma, CMV |
| 88 | Brazil | Adult | 34 | Male | Positive | Disseminated Toxoplasmosis | CMV |
| 89 | Brazil | Adult | 22 | Female | Positive | Pneumonia (CMV) | Toxoplasma, Pneumocystis |
| 90 | Brazil | Adult | 27 | Male | Positive | Disseminated histoplasmosis |  |
| 91 | Brazil | Adult | 43 | Male | Positive | Disseminated Cryptococcosis | S. pneumoniae |
| 92 | Brazil | Adult | 52 | Male | Positive | Pneumonia (no microorganism) |  |
| 93 | Brazil | Adult | 43 | Male | Positive | Pneumonia (CMV) |  |
| 94 | Brazil | Adult | 39 | Female | Positive | Miliary tuberculosis |  |
| 95 | Brazil | Adult | 35 | Female | Positive | Pulmonary tuberculosis |  |
| 96 | Brazil | Adult | 46 | Female | Positive | Disseminated Toxoplasmosis | Bacterial pneumonia (Pseudomonas) |
| 97 | Brazil | Adult | 36 | Female | Positive | Cerebral Toxoplasmosis | CMV |
| 98 | Brazil | Adult | 78 | Male | Positive | Miliary tuberculosis | Pneumocystis, toxoplasma, tuberculosis |
| 99 | Brazil | Adult | 30 | Male | Positive | Cerebral Toxoplasmosis | K. pneumoniae |
| 100 | Brazil | Adult | 33 | Male | Positive | Pneumonia (no microorganism) |  |
| 101 | Brazil | Adult | 46 | Male | Positive | Cerebral Toxoplasmosis | Hypertension |
| 102 | Brazil | Adult | 31 | Male | Positive | Sepsis (S. pneumoniae) | Toxoplasma |
| 103 | Brazil | Adult | 27 | Female | Positive | Disseminated histoplasmosis | Toxoplasma |
| 104 | Brazil | Adult | 38 | Male | Positive | Disseminated Cryptococcosis | CMV |
| 105 | Brazil | Adult | 39 | Male | Positive | Pneumonia (Pneumocystis) | CMV |
| 106 | Brazil | Adult | 34 | Male | Positive | Disseminated histoplasmosis | Pneumonia (Pneumocystis) |
| 107 | Brazil | Adult | 31 | Male | Positive | Miliary tuberculosis | Cytomegalovirus |
| 108 | Brazil | Adult | 30 | Male | Positive | Disseminated cytomegalovirus | Tuberculosis |
| 109 | Brazil | Adult | 34 | Male | Positive | Cerebral Cryptococcosis | Toxoplasma, Histoplasma |
| 110 | Brazil | Adult | 50 | Male | Positive | Pneumonia (cytomegalovirus) | K. pneumoniae |
| 111 | Mozambique | Maternal death | 30 | Female | Positive | Cerebral Cryptococcosis |  |
| 112 | Mozambique | Maternal death | 27 | Female | Positive | Sepsis (Mycoplasma) | Tuberculosis |
| 113 | Mozambique | Maternal death | 28 | Female | Positive | Cerebral malaria |  |
| 114 | Mozambique | Maternal death | 27 | Female | Positive | Sepsis (Enterobacteria) |  |
| 115 | Mozambique | Maternal death | 39 | Female | Positive | Obstetric hemorrhage | Tuberculosis, CMV |
| 116 | Mozambique | Maternal death | 25 | Female | Positive | Miliary tuberculosis | CMV |
| 117 | Mozambique | Maternal death | 25 | Female | Positive | Pyelonephritis (no microorganism) | Cardiomyopathy |
| 118 | Mozambique | Maternal death | 23 | Female | Positive | Cerebral malaria |  |
| 119 | Mozambique | Maternal death | 21 | Female | Positive | Disseminated Cryptococcosis |  |
| 120 | Mozambique | Maternal death | 37 | Female | Positive | Miliary tuberculosis | Bacterial pneumonia (E. coli), CMV |
| 121 | Mozambique | Maternal death | 28 | Female | Positive | Hepatic failure, unspecified | Bacteria (Enterobacteria) |
| 122 | Mozambique | Maternal death | 34 | Female | Positive | Sepsis (streptococcus) | Bacterial (Streptococcus) pneumonia |
| 123 | Mozambique | Maternal death | 27 | Female | Positive | Hepatic failure, unspecified |  |
| 124 | Mozambique | Maternal death | 27 | Female | Positive | Sepsis (o microorganism) |  |
| 125 | Mozambique | Maternal death | 32 | Female | Positive | Obstetric hemorrhage |  |
| 126 | Mozambique | Maternal death | 37 | Female | Positive | Eclampsia |  |
| 127 | Mozambique | Maternal death | 28 | Female | Positive | Cerebral malaria |  |
| 128 | Mozambique | Maternal death | 25 | Female | Positive | Pulmonary tuberculosis |  |
| 129 | Mozambique | Maternal death | 30 | Female | Positive | Disseminated Burkitt lymphoma |  |
| 130 | Mozambique | Maternal death | 34 | Female | Positive | Pneumonia (Staphylococcus) | Kaposi's sarcoma |
| 131 | Mozambique | Maternal death | 30 | Female | Positive | Pneumonia (no microorganism) |  |
| 132 | Mozambique | Maternal death | 35 | Female | Positive | Obstetric hemorrhage | CMV |
| 133 | Mozambique | Maternal death | 24 | Female | Positive | Obstetric hemorrhage |  |
| 134 | Mozambique | Maternal death | 34 | Female | Positive | Disseminated Cryptococcosis | CMV |
| 135 | Mozambique | Maternal death | 31 | Female | Positive | Disseminated Cryptococcosis |  |
| 136 | Mozambique | Maternal death | 19 | Female | Positive | Meningitis (no microorganism) | Tuberculosis, Pneumocystis, CMV |
| 137 | Mozambique | Maternal death | 27 | Female | Positive | Sepsis (no microorganism) |  |
| 138 | Mozambique | Maternal death | 28 | Female | Positive | Obstetric hemorrhage |  |
| 139 | Mozambique | Maternal death | 35 | Female | Positive | Cardiovascular |  |
| 140 | Mozambique | Maternal death | 39 | Female | Positive | Miliary tuberculosis |  |
| 141 | Mozambique | Maternal death | 27 | Female | Positive | Sepsis (no microorganism) |  |
| 142 | Mozambique | Maternal death | 35 | Female | Positive | Sepsis (enterobacteria) | CMV |
| 143 | Mozambique | Maternal death | 25 | Female | Positive | Obstetric hemorrhage |  |
| 144 | Mozambique | Maternal death | 28 | Female | Positive | Pneumonia (no microorganism) |  |
| 145 | Mozambique | Maternal death | 32 | Female | Positive | Miliary tuberculosis | CMV |
| 146 | Mozambique | Maternal death | 36 | Female | Positive | Non-conclusive |  |
| 147 | Mozambique | Pediatric | 7 | Male | Positive | Disseminated Burkitt lymphoma |  |
| 148 | Mozambique | Pediatric | 4m | Male | Positive | Disseminated cytomegalovirus |  |
| 149 | Mozambique | Pediatric | 3m | Female | Positive | Pneumonia (Pneumocystis) |  |
| 150 | Mozambique | Pediatric | 1 | Male | Positive | Pneumonia (no microorganism) |  |
| 151 | Mozambique | Pediatric | 8 | Female | Positive | Tetanus |  |
| 152 | Mozambique | Pediatric | 1 | Male | Positive | Sepsis (S. pneumoniae) |  |
| 153 | Mozambique | Pediatric | 7 | Male | Positive | Sepsis (S. pneumoniae) |  |
| 154 | Mozambique | Pediatric | 2 | Male | Positive | Meningitis (S. pneumoniae) |  |
| 155 | Mozambique | Pediatric | 6m | Female | Positive | Pneumonia (CMV) |  |
| 156 | Mozambique | Pediatric | 6m | Male | Positive | Pneumocystis |  |
| 157 | Mozambique | Pediatric | 9 | Female | Positive | Disseminated CMV |  |
| 158 | Mozambique | Pediatric | 9m | Male | Positive | Peritonitis (no microorganism) |  |
| 159 | Mozambique | Pediatric | 1 | Female | Positive | Pneumonia (CMV) |  |
| 160 | Mozambique | Pediatric | 4 | Male | Positive | Pneumonia (S. pneumoniae) |  |
| 161 | Mozambique | Pediatric | 14 | Female | Positive | Acute lymphoblastic leukaemia |  |
| 162 | Mozambique | Pediatric | 2 | Male | Positive | Sepsis (E. coli) |  |
| 163 | Mozambique | Pediatric | 8m | Male | Positive | Pneumonia (CMV) |  |
| 164 | Mozambique | Neonate | 1m | Male | Positive | Disseminated CMV |  |

CMV: cytomegalovirus; HBV: Hepatitis B virus; HSV-1: herpes symplex virus
